# Respiratory pulsations affect fontan connection power loss: using real time velocity mapping to improve the accuracy of computational simulations

**DOI:** 10.1186/1532-429X-17-S1-O95

**Published:** 2015-02-03

**Authors:** Elaine Tang, Zhenglun (Alan) Wei, Kevin K Whitehead, Alessandro Veneziani, Mark A Fogel, Ajit P Yoganathan

**Affiliations:** 1 Chemical Engineering, Georgia Institute of Technology, Atlanta, GA, USA; 2 Biomedical Engineering, Georgia Institute of Technology, Atlanta, GA, USA; 3 Division of Cardiology, Children's Hospital of Philadelphia, Philadelphia, PA, USA; 4 Mathematics & Computer Science, Emory University, Atlanta, GA, USA

## Background

Total cavopulmonary connection (TCPC) hemodynamics has been hypothesized to be associated with long-term complications in single ventricle heart defect patients. Breath-holding or averaged free-breathing segmented phase contrast magnetic resonance imaging (PC-MRI) has been commonly used for the boundary conditions in numerical simulations to evaluate TCPC hemodynamics. However, the impact of ignoring respiration in the evaluation is not fully understood.

## Methods

Nine patients with TCPC were included. Real-time PC-MRI images were acquired under resting free-breathing (FB) and breath-holding (BH) conditions at superior and inferior vena cava (SVC and IVC). Patient specific 3D TCPC anatomies were reconstructed from transverse CMR images. Computational fluid dynamics (CFD) simulations were performed using caval flow waveforms derived from real-time PC-MRI as inlet boundary conditions. A Windkessel three-element model was applied at the outlets to model the downstream vasculature. Average flow rates and pulsatility indices ([maximum-minimum]/average flow rate) under these two conditions throughout the duration of one respiratory cycle were compared. TCPC power loss was quantified and qualitative flow structure within the TCPC was compared between FB and BH conditions. Lagrangian particle tracking was performed for both conditions to quantify particle washout time.

## Results

**Figure 1:**
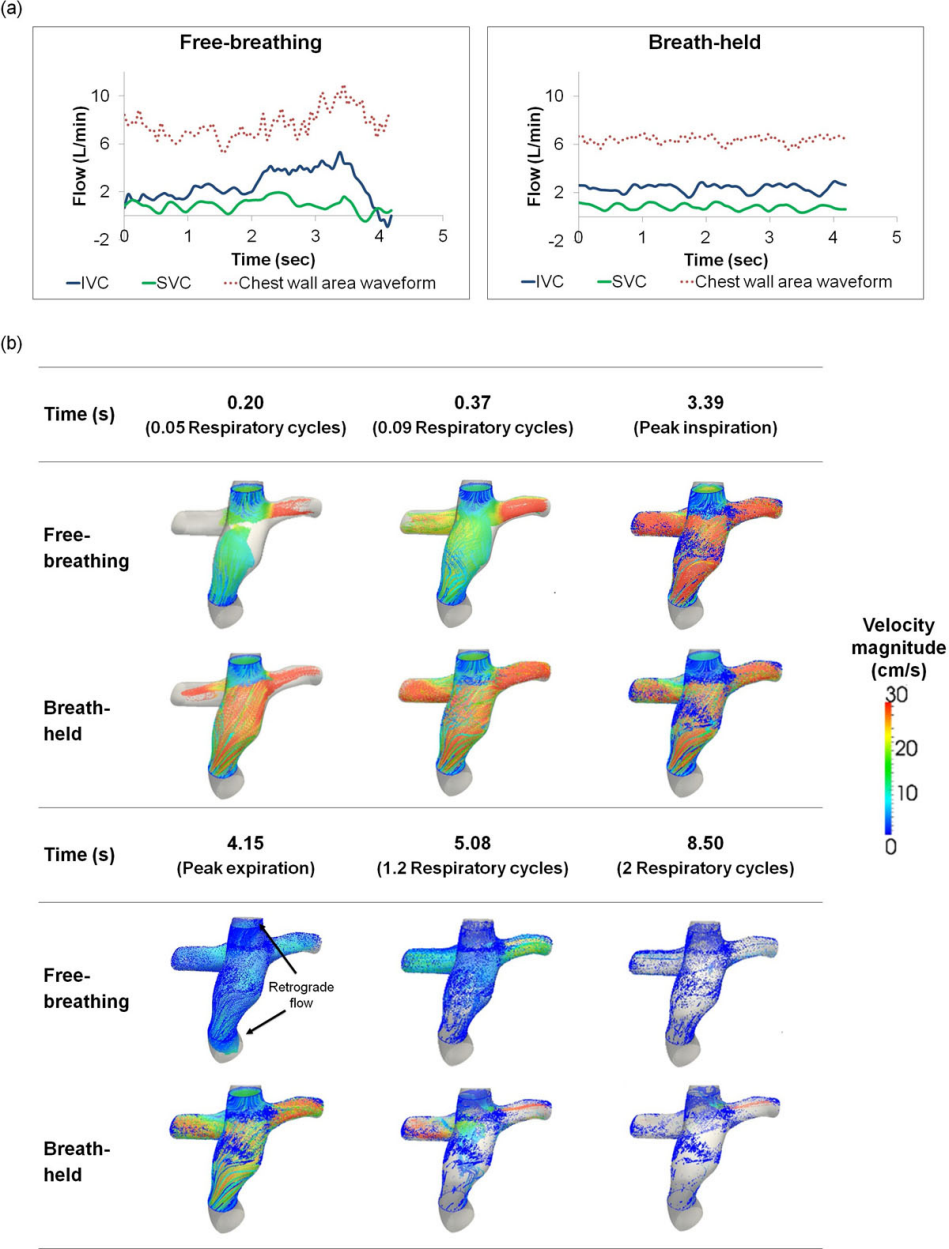
(a) Resting flow waveforms of IVC and SVC of Patient 1 at FB and BH conditions for 1 respiratory cycle (4 cardiac cycles for BH), along with the respiratory cycle determined by tracking the chest wall motion from the IVC MRI images; (b) Particle tracking results within the TCPC under simulated FB and BH conditions for Patient 1 at various time points. Particles were seeded at the IVC and SVC for 1 respiratory cycle, and were allowed to advect for additional 5 respiratory cycles.

The average vessel flow rates at the IVC and SVC were found to be 3.3±0.9L/min and 1.9±1.1L/min at FB compared to 3.1±1.1 L/min and 1.4±1.1 L/min during BH condition. Pulsatility indices were on average 211%±124% (IVC) and 224%±95% (SVC) during FB, compared to 102%±60% (IVC) and 176%±90% (SVC) during BH. Taking CFD results of Patient 1 as example, inspiration led to higher anterograde flow compared to breath-held condition, and expiration causes retrograde flow; together, they result in differences in flow structure between FB and BH conditions. From the particle tracking results, more particles remain within the TCPC volume after 2 respiratory cycles in the FB condition compared to BH (Figure 1), highlighting the longer particle washout time in the FB condition. Higher power loss across the connection was also observed in the FB condition in Patient 1 (Table 1).

**Table 1:** Connection power loss of Patient 1 evaluated using FB and BH flow conditions

		Free-breathing	Breath-held
IVC vessel flow (L/min)	Time-averaged	2.51	2.40
	
	PI	249%	56%

SVC vessel flow (L/min)	Time-averaged	0.88	0.82
	
	PI	276%	110%

Average power loss (mW)	(i) Using time-averaged flow	4.78	4.17
	
	(ii) Using pulsatile flow	7.56	4.28
	
	% Increase from (i) to (ii)	58%	3%

Particle washout time*		1.28	1.08

*Defined as number of respiratory cycles necessary for 95% of the mass-less particles seeded at the inlet to exit the TCPC volume.

## Conclusions

Despite minimal impact on net flows, respiration has considerable impact on TCPC hemodynamics. Vessel flow waveforms acquired with FB condition have higher flow pulsatility. This translates into qualitative differences in flow structure within the connection, and increases in TCPC power loss. The importance of respiratory effects is highlighted, and potential error of calculation of energy dissipation by using BH acquisitions is demonstrated.

## Funding

NIH R01 HL098252-01.

